# Toxicity study of ethanolic stem bark extract of *Xylopia aethiopica* on fertility indices of male rats: An experimental study

**DOI:** 10.18502/ijrm.v13i4.6889

**Published:** 2020-04-30

**Authors:** Ben Enoluomen Ehigiator, Elias Adikwu

**Affiliations:** ^1^Department of Pharmacology and Toxicology, Faculty of Pharmacy, Madonna University, Nigeria.; ^2^Department of Pharmacology and Toxicology, Faculty of Pharmacy, Niger Delta University, Nigeria.

**Keywords:** Xylopia aethiopica, Toxicity, Sperm, Hormone, Rat.

## Abstract

**Background:**

The uses of toxicologically unscreened plants to enhance fertility can be associated with adverse consequences.

**Objective:**

This study aimed to evaluate the effect of the ethanolic stem back extract of *X. aethiopica* (EEXA) on the fertility indices of male albino rats.

**Materials and Methods:**

Sixty male albino rats (weighing 200-250 gr) were grouped and administered by gavage with 200-800 mg/kg of EEXA daily for 15, 30, and 60 days. After the administration of EEXA, the rats were weighed and sacrificed. Blood samples were collected, serum samples were extracted, and evaluated for testosterone, follicle stimulating hormone, prolactin, estradiol, luteinizing hormone and progesterone levels. The testes, epididymis, and prostrate were harvested, weighed and testes were evaluated for sperm parameters.

**Results:**

Significant increase in body weight (p = 0.02) with significant decreases in testes (p = 0.01), epididymis (p = 0.01), and prostate (p = 0.02) weights occurred in rats administered with EEXA when compared to the control group. Significant (p < 0.001) dose and time- dependent decreases in sperm count, volume, motility, and normal morphology were obtained in rats administered with EEXA when compared to the control group. However, there were no significant (p > 0.05) effects on sperm pH when compared to control. Furthermore, luteinizing hormone, follicle stimulating hormone, and testosterone levels were significantly decreased whereas serum prolactin, estradiol, and progesterone levels were significantly increased in a dose-dependent fashion in rats administered with EEXA when compared to the control group.

**Conclusion:**

The findings in this study showed that the use of *X. aethiopica* may be detrimental to male reproduction function.

## 1. Introduction

Sexual relationships are highly imperative in humans for procreation. Sexual dysfunction which is a repeated inability to achieve normal sexual intercourse affects sexual relationships in humans. It can manifest in various forms like retrograded or retarded ejaculation, premature ejaculation, and erectile dysfunction, etc (1). The aforementioned forms of sexual dysfunctions have led to increase rate of divorce in marriages (2). Sexual dysfunction has necessitated the need for the development of various aphrodisiacs, vacuum erection pumps, and surgery to aid erection, improve sperm viability, and other spermatic parameters (3). The search for chemical isolates from medicinal plants to treat sexual dysfunction has increased, probably due to reduced cost, less side effects, and availability. Therefore, the need for safety assessment of medicinal plants with aphrodisiac potential is imperative (4, 5).

*Xylopia aethiopica* (X *aethiopica*) is an evergreen tree that grows up to 20 m tall and 60-70 cm in diameter. It has a straight stem with slightly stripped and smooth bark. *X aethiopica* is a medicinal plant that has recently attracted lots of interest due to its application in folk medicine across the globe (6). It belongs to the Annonaceae family and is commonly found in lowland rainforest and moist fringe forest in the savannah zone of Africa (7). It is used for the treatment of dyspepsia, cough, fever, neuralgia, dysentery, bronchitis, and boils. It is also used as flavouring agent in food and fragrance in cosmetic industry (8). Its fruits, leaf, and stem bark have antibacterial, antifungal, antidiabetic, anti-inflammatory, and antihypertensive properties (8). Furthermore, in addition to its use in the treatment of several diseases, it is also used in folk medicine for fertility enhancement in both males and females which requires validation. Few studies using animal model have reported the effects of the leaves, fruits, and roots of X *aethiopica* on the markers of reproductive function (9), however, there is no literature on the dose and time-dependent profile of the stem back of *X. aethiopica *on reproduction indices. In view of the quest to bridge this information gap, the present study deem it necessary to evaluate the dose and time-dependent effects of the ethanolic stem bark extract of *X. aethiopica* (EEXA) in a rat model. Information from this study may play a critical role in the ethno-medicinal application of *X aethiopica *in fertility enhancement in males.

## 2. Materials and Methods

### Plant

#### Collection and identification of plant material

*X. aethiopica *stem was sourced from Ama Hausa in Imo State, Nigeria. The stem was identified in Benin City, Edo State at the Federal Ministry of Environment and Forestry Research Institute of Nigeria.

#### Preparation of plant extract

*X. aethiopica stem bark *was air dried for 48 h and chopped into smaller pieces using a manual grinder. Thereafter 900 gr was weighed and macerated in ethanol (2000 mL) for 72 h with intermittent shaking. The extract was filtered and concentrated using a rotary evaporator. The extract produced a yield of 50 gr which was used for the study.

#### Phytochemical analysis of *Xylopia aethiopica* stem back

Anthraquinone, combine anthraquinone, steroids, tannins, glycosides, saponins, alkaloids, flavonoids, and terpenoids were evaluated as described by Harbourne (10) and Trease and Evans (11).

### Animal

Sixty 13-week-old male albino rats weighing 200-250gr were purchased from the Animal Breeding Facility of the Department of Pharmacology and Toxicology, Faculty of Pharmacy, Madonna University, Elele, Nigeria. The rats were placed in gauzed cages in the Department of Pharmacology and Toxicology, Faculty of Pharmacy, Madonna University, Elele, Nigeria. The rats had *ad* *libitum* access to feed and water and maintained under standard conditions. The rats were acclimatized for 2 wk before the study began.

### Acute toxicity test

The acute toxicity test was carried out in 2 phases using Lorke's method (12). In phase 1, nine rats were divided into three groups (n = 3/each). The rats in these groups were orally administered with 10, 100, and 1000 mg/kg of EEXA. The rats were observed for 24 h for behavioral changes and mortality. In phase 2, three rats were divided into three groups (n = 1/each) and were orally administered with 1500, 2500, and 5000 mg/kg of EEXA. The rats were observed for 24 h for changes in behavior and mortality. This study observed no mortality in any of the groups which shows that EEXA has a wide therapeutic index.

### Grouping of rats and EEXA administration

A total of sixty rats were divided into 4 groups (n = 15/each)

• Rats in group 1 served as the control group and took food and water only for 15, 30 and 60 days.

• Rats in group 2 were administered orally with 200 mg/kg of EEXA daily for 15, 30 and 60 days.

• Rats in group 3 were administered orally with 400 mg/kg of EEXA daily for 15, 30 and 60 days.

• Rats in group 4 were administered orally with 800 mg/kg of EEXA daily for 15, 30 and 60 days.

### Animal sacrifice and collection of samples

The rats were weighed and sacrificed with the aid of diethyl ether at the end of EEXA administration. Blood samples were collected, allowed to clot, and centrifuged at 2000 rpm for 20 min. Serum samples were extracted and evaluated for reproductive hormones. The rats were dissected and testes, epididymis, and prostate were removed, fat and connective tissues were cleared and weighed. Testes were evaluated for sperm parameters.

### Evaluation of sperm parameters and reproductive hormones

The epididymides were dissected, weighed, and collected in a Petri dish containing physiological saline. The epididymides were incubated at 37 C for 30 min to allow the movement of spermatozoa out of the epididymal tubules. The resulting suspension was collected in a clean graduated tube through a nylon mesh to remove debris and fibrous tissues from the sperm suspension. The collected sperm suspension was used for the evaluation of sperm parameters. The test for sperm viability, morphology and volume were performed as described by the World Health Organization (13). The assessments of sperm count and motility were performed according to the method described by Freund and Carol (14). The levels of serum testosterone, estradiol, prolactin, progesterone, luteinizing hormone, and follicle-stimulating hormones were evaluated using tube-based enzyme-linked immunoassay (EIA) technique.

### Ethical consideration 

This study was approved by the Research Ethics Committee of the Department of Pharmacology and Toxicology, Faculty of Pharmacy, Madonna University, Elele, Nigeria (Pharm/No27/2017). This study was performed in accordance with the guidelines approved by the Research Ethics Committee, the Department of Pharmacology and Toxicology, Faculty of Pharmacy, Madonna University, Elele, Nigeria.

### Statistical analysis

Data were analyzed using Two-way analysis of variance (ANOVA) (graph pad prism version 5) followed by Tukey's post hoc test. Data are expressed as mean ± SEM. Differences between means were regarded as significant at p < 0.05; 0.01; 0.001.

## 3. Results

No deaths were observed in phase 1 and 2 of the acute toxicity evaluation of EEXA (Table I). The phytochemical screening of EEXA showed high concentrations of alkaloids, saponins, and tannins and moderate concentrations of flavonoids, steroids, and cardiac glycosides (Table I). The administration of 200-800mg/kg of EEXA for 15 and 30 days did not produce significant (p > 0.05) effects on body, testicular, epididymis, and prostate weights when compared to the control group. However, significant increase in the body weight (p = 0.03) with significant decreases in testicular (p = 0.01), epididymis (p = 0.01), and prostate (p = 0.02) weights were observed in rats administered with 800 mg/kg of EEXA for 60 days when compared to the control group (Table II). Furthermore, dose and time-dependent decreases in sperm count, motility, normal morphology, volume, and viability were observed in rats administered with 200-800 mg/kg of EEXA for 15, 30, and 60 days when compared to the control group (Tables III-IV). Significant decreases in sperm viability (p = 0.02), volume (p = 0.03), normal morphology (p = 0.02), sperm count (p = 0.02), and motility (p = 0.03) were observed in rats administered with 400mg/kg of EEXA for 30 days when compared to the control group (Table III). However, significant decreases in normal morphology (p = 0.001), sperm count (p = 0.001), and motility (p = 0.001) were observed after 60 days of the administration of 800 mg/kg of EEXA when compared to the control group (Table III). In contrast, treatment with EEXA did not produce significant effect on sperm pH when compared to the control group (p = 0.09) (Table IV). Furthermore, this study observed significant increases in serum prolactin, progesterone and estradiol hormones with significant decreases in serum testosterone, luteinizing, and follicle stimulating hormones in a dose and time-dependent fashion after the administration of 200 and 400 mg/kg of EEXA for 15, 30, and 60 days respectively when compared to the control group (Tables IV and V). Significant (p < 0.001) increases in serum prolactin, estradiol, and progesterone levels with significant decreases in serum testosterone, luteinizing, and follicle stimulating hormones were observed in rats administered with 800 mg/kg of EEXA for 60 days when compared to the control group (Tables IV and V).

**Table 1:** Phytochemical screening and acute toxicity evaluation of the ethanolic stem bark extract of *Xylopia aethiopica*


**Constituents**		**Present/Absent**	**Interpretation**
**Alkaloids**		+++	High concentration
**Saponins**		+++	High concentration
**Flavonoids**		++	Moderate concentration
**Tannins**		+++	High concentration
**Free anthraquinone**		–	Absent
**Combine anthraquinone**		–	Absent
**Terpenes**		+ Trace concentration
**Steroids**		++	Moderate concentration
**Cardiac glycosides**		++	Moderate concentration
**Acute toxicity test (LD-50)**			
**Dose mg/kg**		**No. of rat per group**	**Death per group**
**PHASE 1**		
	**10**	3	0/3
	**100**	3	0/3
	**1000**	3	0/3
**PHASE 2**			
	**1500**	1	0/1
	**2500**	1	0/1
	**5000**	1	0/1

**Table 2:** Effects of *Xylopia aethiopica *on body and organ weights of albino rats


**Dose**	**FBW (g)**	**ATW (g)**	**RTW (%)**	**AEDM (g)**	**REDM (%)**	**APW (g)**	**RPW (%)**
**Control**	250.6 ± 3.33	1.82 ± 0.07	0.65 ± 0.03	0.73 ± 0.05	0.29 ± 0.09	0.90 ± 0.05	0.36 ± 0.09
**200 mg/kg**	254.0 ± 14.0	1.74 ± 0.05	0.59 ± 0.01	0.67 ± 0.02	0.26 ± 0.06	0.83 ± 0.02	0.33 ± 0.07
**400 mg/kg**	270.5 ± 12.8	1.60 ± 0.02	0.58 ± 0.09	0.59 ± 0.06	0.22 ± 0.04	0.50 ± 0.05*	0.19 ± 0.01*
**800 mg/kg**	320.3 ± 12.2*	1.31 ± 0.09*	0.38 ± 0.07*	0.35 ± 0.09*	0.11 ± 0.08*	0.41 ± 0.07*	0.13 ± 0.09*
FBW: Final body weight; ATW: Absolute testicular weight; RTW: Relative testicular weight; AEDM: Absolute epididymis weight; REDM: Relative epididymis weight; RPW: Relative prostate weight; APW: Absolute prostate weight Values represent Mean ± SEM (n = 5) using two-way ANOVA test, followed by Tukey's post hoc test *Significant difference when compared to control at p < 0.05							

**Table 3:** Effects of *Xylopia aethiopica *on sperm viability, morphology, count and motility of albino rats


**Durations**		**15 days**	**30 days**	**60 days**
**Sperm viability (%)**				
	**Control**	65.3 ± 4.41	65.7 ± 5.50	67.3 ± 6.31
	**200 mg/kg**	63.0 ± 6.22	61.8 ± 5.30	40.7 ± 5.00*
	**400 mg/kg**	62.1 ± 5.20	50.6 ± 6.00*	31.3 ± 4.06**
	**800 mg/kg**	43.7 ± 4.49*	40.5 ± 5.47**	25.3 ± 5.33***
**Sperm morphology (%)**				
	**Control**	71.6 ± 5.33	72.7 ± 6.61	75.8 ± 4.22
	**200 mg/kg**	70.3 ± 6.00	60.0 ± 5.30	40.7 ± 5.30*
	**400 mg/kg**	67.5 ± 7.22	50.3 ± 5.00*	35.3 ± 6.00**
	**800 mg/kg**	52.3 ± 5.06*	45.7 ± 4.47**	24.8 ± 5.47***
**Sperm count (x10${}^{6}$)**				
	**Control**	70.9 ± 6.34	71.3 ± 5.20	70.7 ± 6.11
	**200 mg/kg**	67.1 ± 4.00	60.0 ± 4.63	50.1 ± 4.63*
	**400 mg/kg**	60.7 ± 5.21	51.5 ± 6.34*	37.5 ± 6.34**
	**800 mg/kg**	55.3 ± 4.32*	45.0 ± 5.15**	30.0 ± 5.15***
**Sperm motility (%)**				
	**Control**	73.3 ± 4.34	75.7 ± 6.53	70.0 ± 6.11
	**200 mg/kg**	70.7 ± 6.17	66.1 ± 5.43	50.1 ± 4.63*
	**400 mg/kg**	65.1 ± 5.44	57.7 ± 5.32*	37.7 ± 6.34**
	**800 mg/kg**	51.3 ± 4.32*	42.0 ± 6.00**	30.0 ± 4.32***
Values represent Mean ± SEM (n = 5) using two-way ANOVA followed by Tukey's post hoc test *Significant difference when compared to control at p < 0.05 **Significant difference when compared to control at p < 0.01 ***Significant difference when compared to control at p < 0.001				

**Table 4:** Effects of *Xylopia aethiopica *on sperm volume, Ph, serum testosterone, and luteinizing hormones of albino rats


**Durations**		**15 days**	**30 days**	**60 days**
**Sperm volume (mL)**				
	**Control**	1.48 ± 0.04	1.50 ± 0.04	1.48 ± 0.06
	**200 mg/kg**	1.43 ± 0.06	1.35 ± 0.01	1.30 ± 0.02*
	**400 mg/kg**	1.35 ± 0.02	1.00 ± 0.08*	0.60 ± 0.09**
	**800 mg/kg**	0.90 ± 0.05*	0.65 ± 0.07**	0.35 ± 0.96***
**Sperm PH**				
	**Control**	6.97 ± 0.07	6.90 ± 0.02	6.96 ± 0.05
	**200 mg/kg**	7.00 ± 0.01	6.98 ± 0.07	6.90 ± 0.03
	**400 mg/kg**	6.95 ± 0.04	7.00 ± 0.09	6.86 ± 0.01
	**800 mg/kg**	6.88 ± 0.20	7.10 ± 0.03	7.00 ± 0.05
**Testosterone (µg/mL)**				
	**Control**	2.37 ± 0.01	2.45 ± 0.07	2.39 ± 0.06
	**200 mg/kg**	2.30 ± 0.03	2.33 ± 0.05	1.65 ± 0.02*
	**400 mg/kg**	2.00 ± 0.04	1.51 ± 0.03*	1.01 ± 0.07**
	**800 mg/kg**	1.50 ± 0.09*	1.00 ± 0.01**	0.54 ± 0.06***
**Luteinizing hormone (µg/mL)**				
	**Control**	1.97 ± 0.07	1.88 ± 0.01	1.92 ± 0.07
	**200 mg/kg**	1.92 ± 0.06	1.64 ± 0.04	1.32 ± 0.05*
	**400 mg/kg**	1.78 ± 0.09	1.33 ± 0.07*	0.91 ± 0.04**
	**800 mg/kg**	1.50 ± 0.01*	1.02 ± 0.03**	0.4 ± 0.01***
All data presented as Mean ± SEM (n = 5/each group). Two-way ANOVA test, followed by Tukey’s post hoc test. *Significant difference when compared to control at p < 0.05; **Significant difference when compared to control at p < 0.01; ***Significant difference when compared to control at p < 0.001				

**Table 5:** Effects of *Xylopia aethiopica *on serum follicle-stimulating, prolactin, progesterone, and estradiol hormones of albino rats


**Durations**	**15 days**	**30 days**	**60 days**
**Follicle-stimulating hormone (µg/mL)**			
**Control**	17.6 ± 1.01	16.9 ± 0.04	18.6 ± 1.55
**200 mg/kg**	16.1 ± 0.32	14.7 ± 1.74	10.7 ± 1.32*
**400 mg/kg**	15.7 ± 1.30	11.0 ± 0.54*	0.64 ± 0.01**
**800 mg/kg**	11.0 ± 1.00*	0.61 ± 0.03**	0.32 ± 0.03***
**Prolactin (µg/mL)**			
**Control**	2.60 ± 0.01	2.81 ± 0.02	2.63 ± 0.05
**200 mg/kg**	2.85 ± 0.22	2.90 ± 0.07	3.48 ± 0.06*
**400 mg/kg**	3.10 ± 0.18	3.49 ± 0.02*	4.36 ± 0.01**
**800 mg/kg**	3.48 ± 0.06*	4.39 ± 0.06**	6.84 ± 0.29***
**Progesterone (µg/mL) **			
**Control**	2.57 ± 0.01	2.40 ± 0.04	2.51 ± 0.43
**200 mg/kg**	2.60 ± 0.04	2.80 ± 0.22	3.42 ± 0.12*
**400 mg/kg**	2.75 ± 0.02	3.57 ± 0.23*	4.51 ± 0.31**
**800 mg/kg**	3.20 ± 0.01*	4.30 ± 0.01**	6.94 ± 0.02***
**Estradiol (µg/mL)**			
**Control**	18.6 ± 2.51	19.2 ± 2.00	18.6 ± 2.25
**200 mg/kg**	17.0 ± 1.04	20.64 ± 0.24	25.4 ± 3.42*
**400 mg/kg**	20.1 ± 2.00	26.0 ± 3.86*	35.7 ± 3.00**
**800 mg/kg**	25.9 ± 2.22*	34.6 ± 3.77**	42.9 ± 4.21***
All data presented as Mean ± SEM (n = 5/each group) Two-way ANOVA test, followed by Tukey’s post hoc test *Significant difference when compared to control at p < 0.05; **Significant difference when compared to control at p < 0.01; ***Significant difference when compared to control at p < 0.001			

## 4. Discussion 

The increasing cost of synthetic aphrodisiacs has created a scene for increasing use of alternative sources which include plants to boost fertility. However, the uses of toxicologically unscreened plants to enhance fertility are without some adverse consequences because the reproductive system is sensitive to chemical insult; any damage can result in reproductive failure or infertility (15, 16). This study assessed the effects of the ethanolic stem back extract of EEXA on sperm indices and reproductive hormones of male albino rats. No rat died in the acute toxicity evaluation of EEXA which showed it has a wide safety margin. The phytochemical screening of EEXA showed high concentrations of alkaloids, saponins and tannins whereas moderate concentrations of flavonoids, steroids and cardiac glycosides were found. This observation is similar to the findings reported when extracts from different parts of *X aethiopica* were phytochemically screened (17). In this study, the testicular insult observed in rats administered with EEXA was marked by decreases in testicular, epididymis and prostate weights. Similarly, Woode and others reported decreases in reproductive organ weights in Wistar rats treated with methanolic extract of the dry fruits of *X* *aethiopica* (18). The significant decreases in testicular weight and other accessory organs of rats observed in this study may be attributed to decreases in testosterone levels (19). In experimental animal studies decreases in sperm count, motility, viability and volume are valid indices for male infertility assessment (20, 21). Among these parameters, sperm motility is often used as a marker of chemical-induced testicular toxicity while sperm morphology assessment has been used as a deferential tool for diagnosing infertility among men (22). The harmful effect of EEXA on sperm parameters observed in this study was characterized by dose and time-dependent decreases in sperm motility, count, volume, viability and normal morphology which are signs of infertility. Similarly, Nwangwa reported decreases in sperm parameters in rats administered with the methanolic extract of the dry fruits of X *aethiopica* (23). The observed decreases in sperm vitality and motility may be attributed to the interference of EEXA with the energy production process required for sperm vitality and motility (24). The decreases in sperm count and volume may be due to the inhibitory effects of EEXA on seminiferous tubules and Leydig cells responsible for the syntheses of sperm and testosterone respectively (25). Furthermore, studies have shown that reproductive hormones are essential for the development of germ cells. The complete development of male germ cell depends on the cordial endocrine interplay of hypothalamus, pituitary and the testis (26). Follicle stimulating hormone stimulates Sertoli cells and facilitates spermatogenesis. Luteinizing hormone stimulates testosterone production in Leydig cells which act on the Sertoli and cells of the seminiferous tubules to facilitate sperm production (27). Xenobiotic associated perturbations of gonadal functions are often characterized by marked alterations in serum reproductive hormones. In the present study, EEXA decreased serum testosterone, luteinizing hormone, and follicle stimulating hormone, whereas prolactin, progesterone and estradiol hormones were increased in a dose and time-dependent fashion. This observation is supported by the findings of Nnodim and others who reported decreased reproductive hormone levels in rats treated with the ethanolic extract of *X. aethiopica* fruits (28). Altered levels of serum estradiol, prolactin, progesterone, testosterone, luteinizing, and follicle stimulating hormones can be correlated with the decrease in sperm quality observed in EEXA-treated rats. The observed decreases in testosterone, luteinizing, and follicle stimulating hormones can be attributed to the inhibitory effect of EEXA on the hypothalamic-pituitary axis which is responsible for the secretion of these hormones.

## 5. Conclusion

The findings in this study showed that the use of *X. aethiopica* may be detrimental to male reproduction function.

##  Conflict of Interest

The authors declare no conflict of interest.
